# Biochemical Effect of Resistance Mutations against Synergistic Inhibitors of RSV RNA Polymerase

**DOI:** 10.1371/journal.pone.0154097

**Published:** 2016-05-10

**Authors:** Jerome Deval, Amy Fung, Sarah K. Stevens, Paul C. Jordan, Tatiana Gromova, Joshua S. Taylor, Jin Hong, Jia Meng, Guangyi Wang, Natalia Dyatkina, Marija Prhavc, Julian A. Symons, Leo Beigelman

**Affiliations:** Alios BioPharma, Inc., part of the Janssen Pharmaceutical Companies, South San Francisco, California, United States of America; Imperial College London, UNITED KINGDOM

## Abstract

ALS-8112 is the parent molecule of ALS-8176, a first-in-class nucleoside analog prodrug effective in the clinic against respiratory syncytial virus (RSV) infection. The antiviral activity of ALS-8112 is mediated by its 5'-triphosphate metabolite (ALS-8112-TP, or 2'F-4'ClCH_2_-cytidine triphosphate) inhibiting the RNA polymerase activity of the RSV L-P protein complex through RNA chain termination. Four amino acid mutations in the RNA-dependent RNA polymerase (RdRp) domain of L (QUAD: M628L, A789V, L795I, and I796V) confer in vitro resistance to ALS-8112-TP by increasing its discrimination relative to natural CTP. In this study, we show that the QUAD mutations specifically recognize the ClCH_2_ group of ALS-8112-TP. Among the four mutations, A789V conferred the greatest resistance phenotype, which was consistent with its putative position in the active site of the RdRp domain. AZ-27, a non-nucleoside inhibitor of RSV, also inhibited the RdRp activity, with decreased inhibition potency in the presence of the Y1631H mutation. The QUAD mutations had no effect on the antiviral activity of AZ-27, and the Y1631H mutation did not significantly increase the discrimination of ALS-8112-TP. Combining ALS-8112 with AZ-27 in vitro resulted in significant synergistic inhibition of RSV replication. Overall, this is the first mechanistic study showing a lack of cross-resistance between mutations selected by different classes of RSV polymerase inhibitors acting in synergy, opening the door to future potential combination therapies targeting different regions of the L protein.

## Introduction

Respiratory syncytial virus (RSV) is an RNA virus and a member of the *Paramyxoviridae* family. RSV infection usually lasts 1–2 weeks and results in mild cold-like symptoms in the majority of adults. However, RSV infection can lead to severe lower respiratory infection in vulnerable populations such as infants. In 2005, an estimated 33.8 million episodes of RSV occurred worldwide in children younger than 5 years old, making RSV the leading cause of serious lower respiratory tract infections in children [1,2]. Of these, 2.8–4.8 million severe cases of lower respiratory infection required hospitalization, and an estimated 66,000 to 199,000 deaths occurred, mostly in the developing world [3]. There are no vaccines approved for the prevention of RSV infection. Palivizumab, a monoclonal antibody directed against RSV, is approved only for prophylaxis in order to prevent RSV-related hospitalizations in high-risk children, but its safety and efficacy have not been established for the treatment of RSV. Treatment of infants with severe RSV bronchiolitis is thus supportive, consisting of oxygen therapy, nutrition, and fluids. Furthermore, there are only a few agents in early stages of clinical development for the treatment of RSV infection [4,5]. As such, there is a need for a novel therapeutic that can be used both in the outpatient setting to reduce the severity of infection and prevent possible hospital admissions, and in the hospital setting, to ameliorate the severity of symptoms and duration of time spent in the hospital.

ALS-8176 is a 3′,5′ bisisobutyrate prodrug of 2'F-4'ClCH_2_-cytidine (ALS-8112), which is being developed as an orally administered antiviral therapy for the treatment of infants, children, and adults infected with RSV. In a human challenge study conducted in adult volunteers, ALS-8176 was efficacious against RSV infection [6]. In tissue culture experiments, ALS-8112 and ALS-8176 are potent and highly selective inhibitors of both RSV laboratory adapted A and B strains as well as a range of diverse clinical isolates [7,8]. In addition, both ALS-8112 and ALS-8176 inhibit RSV replication in the sub-genomic replicon system. The ALS-8112 5′-triphosphate metabolite (ALS-8112-TP) is the active form of the drug and is responsible for RSV inhibition. ALS-8112-TP selectively targets the RSV RNA-dependent RNA polymerase (RdRp) activity carried by the L-P protein complex, via a classic chain termination mechanism [8]. Prolonged in vitro replication of RSV A2 for >35 passages in the presence of ALS-8112 selected for mutations within the region of the L gene of RSV encoding the RdRp function [8]. These four amino acid mutations (QUAD: M628L, A789V, L795I, and I796V) were associated with resistance to ALS-8112. In biochemical assays, the presence of the QUAD mutations, once introduced into recombinant L-P enzyme, caused an increase in discrimination of ALS-8112-TP relative to natural CTP. Although the resistance phenotype could be attributed to the 4′-substitution on the sugar moiety of ALS-8112-TP, the molecular basis for nucleotide resistance induced by the QUAD mutations remains elusive. Moreover, the mechanism of resistance of mutations induced by non-nucleotide inhibitors of RSV remains unknown.

In this study, we aimed to 1) better understand the mechanism and specificity of resistance caused by the QUAD mutations, 2) study other potential mutation-induced resistance effects across various classes of inhibitors of RSV L-P polymerase, and 3) measure the antiviral effect of combining nucleoside and non-nucleoside inhibitors of RSV polymerase. We first analyzed the resistance profile of the QUAD RSV L-P against a panel of 2′F-4′-substituted CTP analogs, and found that the resistance level was the highest with 4′-halomethyl derivatives. We also showed that the nucleobase is an important contributor to the loss of potency of related nucleotide analogs. Among the four mutations, only A789V individually caused a significant increase in drug discrimination in the polymerase assay. We also characterized the mechanism of inhibition of AZ-27, a non-nucleoside inhibitor of RSV polymerase that remains active against the QUAD mutant, but loses inhibition potency in the presence of the Y1631H mutation. Our new data indicate that nucleoside and non-nucleoside inhibitors of RSV polymerase have synergistic effects and complementary resistance mutation profiles, and therefore these two classes of compounds could be potentially evaluated in combination therapies.

## Results

### Specificity of QUAD-mediated resistance: changing the sugar moiety

**Fig 1:**
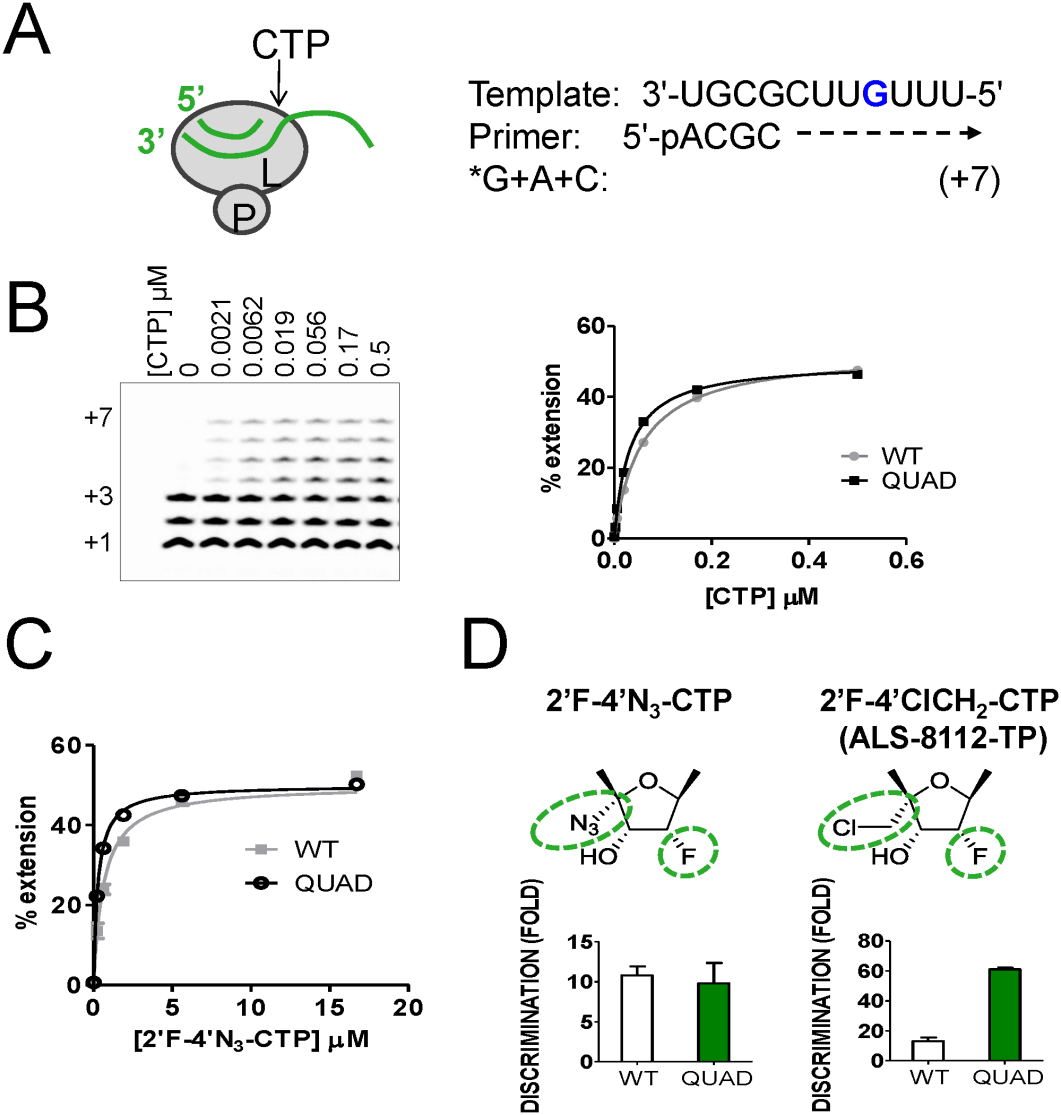
Effect of the QUAD mutations on the discrimination of 2'F-4'N_3_-CTP. (**A**) Principle of the standard primer extension reaction for single CTP incorporation. The L subunit of RSV polymerase binds to the primer/template duplex (green) and carries the RdRp activity. The radiolabeled α^33^P-GTP (*G or *GTP) is incorporated first at position +1, after which ATP and CTP are required to fully extend the primer to the +7 position. (**B**) The RSV L-P proteins (WT and QUAD) were incubated in the presence of *GTP + ATP and increasing concentrations of CTP. Lane 1, no CTP; lane 2, 0.0021 μM; lane 3, 0.0062 μM; lane 4, 0.019 μM; lane 5, 0.056 μM; lane 6, 0.17 μM; and lane 7, 0.5 μM CTP. Product formation was quantified and expressed as percentage primer extension from the +3 position. WT K_m CTP_ = 0.056±0.010 μM (n = 4), and QUAD K_m CTP_ = 0.028±0.005 μM (n = 2) [8]. (**C**) The same experiment was repeated for each enzyme with 2'F-4'N_3_-CTP, resulting in a WT K_m 2'F-4'N3-CTP_ = 0.62±0.042 μM (n = 2), and QUAD K_m 2'F-4'N3 CTP_ = 0.28±0.07 μM (n = 2). (**D**) For each enzyme, the level of discrimination was calculated as K_m CTP analog_ / K_m CTP_, and resistance as QUAD_discrimination_ / WT_discrimination_.

**Fig 2:**
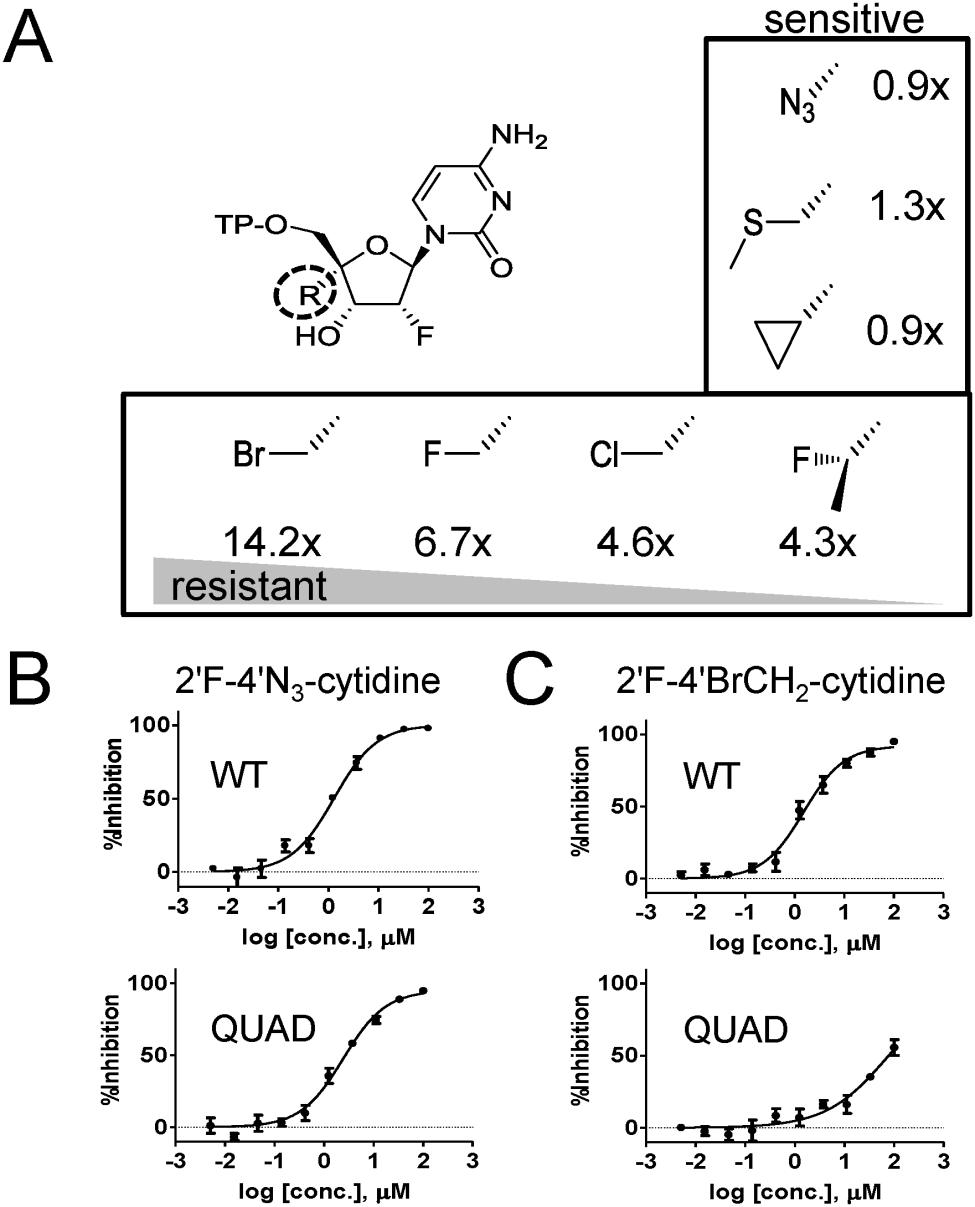
Effect of the QUAD mutations on the discrimination of 2'F-CTP analogs. (**A**) Measurement of resistance level of QUAD RSV L-P against a series of 2'F-CTP analogs with the following substitution at the 4'position: chloromethyl (ClCH_2_), fluoromethyl (FCH_2_), bromomethyl (BrCH_2_), 1-fluoroethyl (FCH_3_CH_2_), azido (N_3_-), cyclopropyl (CH_2_CH_2_CH_2_), and methyl-thio-methyl (CH_3_SCH_2_). For each CTP analog, the level of resistance was calculated as described for Fig 1 (n = 2). (**B**) In vitro inhibition potency of 2'F-4'N_3_-cytidine against the RSV minigenome luciferase-based reporter assay. HEp-2 cells were co-transfected to transiently express the RSV N, P, M2-1 and L proteins containing either the WT or the QUAD mutated L sequence (n = 4). (**C**) Inhibition of luciferase-based WT and QUAD RSV minigenome activity by 2'F-4'BrCH_2_-cytidine (n = 4).

We previously reported resistance to 2'F-4'ClCH_2_-CTP (ALS-8112-TP) by the QUAD mutations in RSV polymerase, and we wanted to know if the same mutations also confer resistance to other 2'F-CTP analogs such as 2'F-4'N_3_-CTP [8]. An in vitro primer extension assay was developed to measure the incorporation efficiency of a single cytidine molecule by recombinant RSV polymerase (Fig 1A). Under this assay format, the incorporation of natural CTP at position +4 by the viral polymerase results in the formation of a +7 fully extended primer (Fig 1B). The four amino acid mutations in the polymerase (QUAD: M628L, A789V, L795I, and I796V) did not result in any decrease in the efficiency of incorporation of natural CTP with a K_m_ value of 0.028±0.005 μM compared to 0.056±0.010 μM for the wild-type (WT) enzyme. Similarly, 2'F-4'N_3_-CTP was efficiently recognized as substrate by the QUAD mutant (K_m_ = 0.28±0.07 μM vs. 0.62±0.042 μM for WT), which resulted in no increase in discrimination compared to the WT enzyme (Fig 1C and 1D). In contrast, our previous results showed a 4.6-fold increase in discrimination of 2'F-4'ClCH_2_-CTP (ALS-8112-TP) associated with the QUAD mutation (Fig 1D [8]). The difference in discrimination results led us to hypothesize that the nucleotide resistance effect associated with the QUAD mutations might depend on the type of substitution at the 4'-position on the nucleotide sugar moiety. To verify the hypothesis we measured potential changes in QUAD discrimination against a series of 2'F-4'-substituted CTP analogs. Overall, we found that chloromethyl (ClCH_2_), fluoromethyl (FCH_2_), bromomethyl (BrCH_2_), and 1-fluoroethyl (FCH_3_CH_2_) at the 4'-position contributed to a resistance level >3-fold, whereas, azido (N_3_-), cyclopropyl (CH_2_CH_2_CH_2_), and methyl-thio-methyl (CH_3_SCH_2_) induced no resistance (Fig 2A).

The contribution of 4'-halomethyl substitutions toward QUAD-mediated resistance was also evaluated in the cell-based RSV minigenome system. In this assay, the transient expression of RSV N, P, M2-1, and L in HEp-2 cells leads to the formation of a functional RSV polymerase complex, and its RdRp activity is monitored using a luciferase-based reporter. In this system, 2'F-4'N_3_-cytidine was equally effective at inhibiting WT and QUAD RSV L polymerase-dependent luciferase activity with half-maximal effective concentrations (EC_50_) of 1.3±0.3 and 2.2±0.5 μM, respectively (Fig 2B). In comparison, 2'F-4'BrCH_2_-cytidine inhibited WT RSV L with an EC_50_ value of 1.6±0.6 μM, but exhibited a 47-fold loss in inhibition potency in the presence of the QUAD mutations (Fig 2C). These results confirm, at the virus replication level, that the effect of the QUAD mutations in L is most pronounced in the presence of 2'F-cytidine analogs containing a 4'-halomethyl. This information could potentially be used to design novel nucleotide analogs that would remain active against the QUAD RSV polymerase.

### Specificity of QUAD-mediated resistance: changing the nucleobase

**Fig 3:**
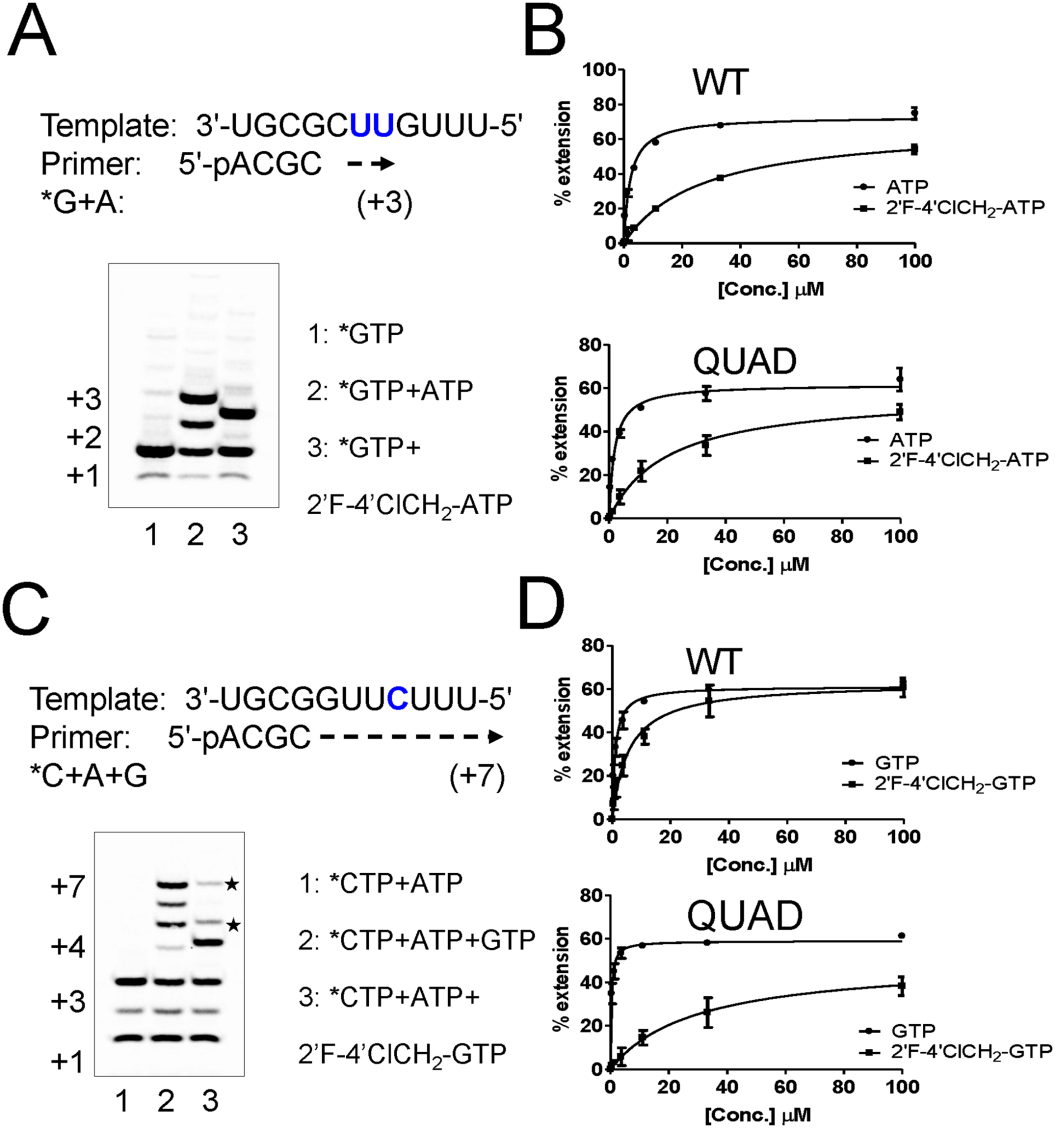
Discrimination of purine analogs by the QUAD mutant. (**A**) Incorporation of 2'F-4'ClCH_2-_ATP by WT L-P and chain termination effect. Lane 1: *GTP only (+1 product), Lane 2: *GTP+ATP (+3 product), Lane 3: *GTP+2'F-4'ClCH_2_-ATP (+2 product). (**B**) The RSV L-P proteins (WT and QUAD) were incubated in the presence of *GTP and increasing concentrations of either ATP or 2'F-4'ClCH_2_-ATP. (**C**) Incorporation of 2'F-4'ClCH_2_-GTP by WT L-P and chain termination effect. The sequence of the standard template was modified to enable single G incorporation at position +4. Lane 1: *CTP+ATP, Lane 2: *CTP+ATP+GTP, Lane 3: *CTP+ATP+2'F-4'ClCH_2_-GTP. (**D**) The RSV L-P proteins (WT and QUAD) were incubated in the presence of *CTP+ATP and increasing concentrations of either GTP or 2'F-4'ClCH_2_-GTP.

We also evaluated the effect on drug resistance resulting from changing the nucleobase on the 2'F-4'ClCH_2_ ribose scaffold from cytosine to adenine and guanine. The standard in vitro primer extension assay was modified slightly to study the incorporation of adenosine triphosphates. In the presence of radiolabeled α^33^P-GTP (*GTP) and natural ATP, the RNA template allowed the incorporation of two consecutive adenosines, extending the primer from the +1 to the +3 position (Fig 3A, **lanes 1 and 2**). When ATP was replaced by 2'F-4'ClCH_2_-ATP, the primer extension only reached the +2 position (Fig 3A, **lane 3**), meaning that 2'F-4'ClCH_2_-ATP acted as an immediate chain terminator of WT RSV polymerase. These experiments were repeated at various nucleotide concentrations to determine their K_m_ values, which resulted in K_m_ = 2.2±0.2 μM for ATP and 27±7.8 μM for 2'F-4'ClCH_2_-ATP. The 12-fold discrimination of 2'F-4'ClCH_2_-ATP by WT RSV polymerase did not significantly increase in the presence of the QUAD mutations (Fig 3B, Table 1).

**Table 1:** Incorporation Efficiency of 2'F-4'ClCH_2_ NTP Analogs.

RSV L-P	NTP	K_m_ (μM)	Discrimination	Resistance
WT	ATP	2.2±0.2		
	2'F-4'ClCH_2_-ATP	27±7.8	12±2.5	
QUAD	ATP	1.8±0.3		
	2'F-4'ClCH_2_-ATP	23.6±5.8	12.9±1.1	1.1
WT	GTP	1.1±0.4		
	2'F-4'ClCH_2_-GTP	5.8±1.4	5.6±0.6	
QUAD	GTP	0.3±0.1		
	2'F-4'ClCH_2_-GTP	31.1±15	94.4±17.7	17

The original RNA template sequence was further modified to monitor single events of guanosine incorporation opposite-C (Fig 3C). In the presence of radiolabeled α^33^P-CTP (*CTP) and natural ATP, the 4-mer primer was extended by WT RSV polymerase to the +3 position (Fig 3C, **lane 1**). The addition of natural GTP to the reaction enabled further extension to the full length +7 position (Fig 3C, **lane 2**). However, replacing GTP by 2'F-4'ClCH_2_-GTP resulted in the appearance of a dominant product at position +4, with two minor products of larger size (marked⋆). This result suggests that 2'F-4'ClCH_2_-GTP did not completely block RNA synthesis. When tested against the WT enzyme, the incorporation of 2'F-4'ClCH_2_-GTP was relatively efficient, with a K_m_ value of 5.8±1.1 μM, resulting in a modest 5-fold discrimination compared to natural GTP (K_m GTP_ = 1.1±0.4 μM) (Fig 3D). However, the discrimination of 2'F-4'ClCH_2_-GTP increased to 94.4-fold in the presence of the QUAD mutations (Fig 3D), which corresponded to a 17-fold resistance level compared to WT (Table 1).

### Role of each residue from the QUAD on nucleotide resistance

**Fig 4:**
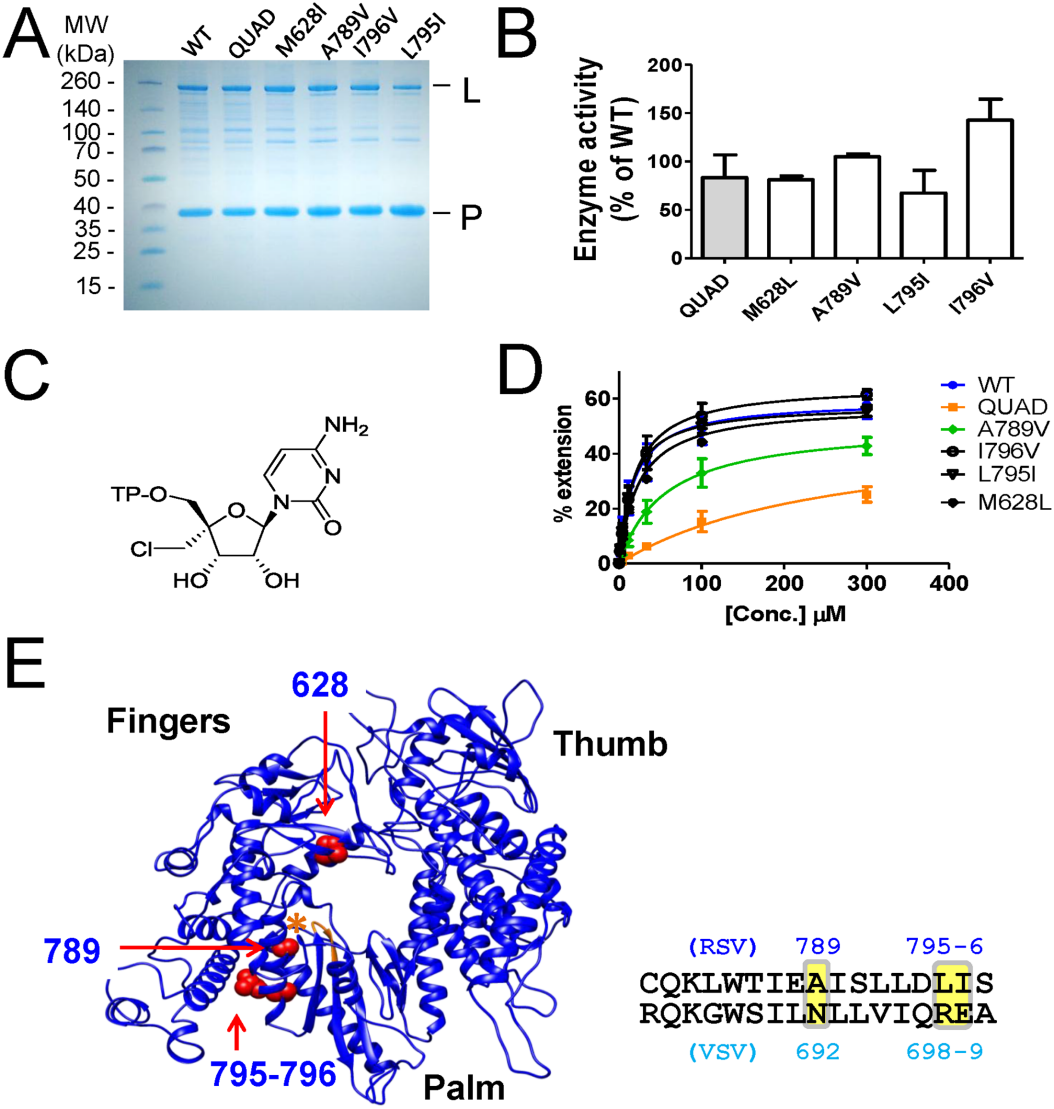
Individual contribution of each of the QUAD mutations. **(A)** SDS PAGE analysis of RSV L-P proteins containing one or four (QUAD) amino acid changes in the L subunit. (**B**) Relative enzyme activity of each protein variant compared to WT RSV L-P, as measured by the total formation of RNA products in the standard primer extension assay. (C) Chemical structure of 4'ClCH_2_-CTP. (**D)** Efficiency of incorporation of 4'ClCH_2_-CTP by WT and mutated RSV L-P. K_m_ values were determined by incubating each enzyme with increasing concentrations of 4'ClCH_2_-CTP, in the presence of *GTP + ATP as described in Fig 1. For 4'ClCH_2_-CTP, WT K_m_ = 19.7±6.7 μM (n = 2), M628L WT K_m_ = 23.1±0.07 μM (n = 2), QUAD K_m_ = 235±77 μM (n = 4), A789 K_m_ = 61.0±26.9 μM (n = 2), L795I K_m_ = 16.5±6.4 μM (n = 2), and I796V K_m_ = 22.0±9.9 μM (n = 2). (E) A homology-based three dimensional structure of the RSV polymerase L protein was generated using RaptorX [10], using the VSV L protein (cyan, PDB = 5a22) as reference. The molecular graphics of the resultant structure (blue) were generated using the UCSF Chimera package [35]. The proposed positioning of residues 628, 789, 795, and 796 from the RSV polymerase model is shown in red, as indicated by arrows. The asterisk (orange) shows the position of the GDNQ catalytic motif.

To assess the discrimination level of each of the QUAD mutations, individual L-P complexes containing a single amino acid change in the L protein were expressed and purified (Fig 4A). Compared to the WT L-P, the variations in overall enzymatic activity were less than 2-fold, with the L795I mutant being the least active most likely because of a notably lower L/P protein ratio (Fig 4A and 4B). To maximize the discrimination effect of each mutation, single nucleotide incorporation experiments were carried out with 4'ClMe-CTP (Fig 4C), a nucleotide analog already known to be strongly discriminated by the QUAD [8]. The K_m_ values for 4'ClMe-CTP were similar between WT (19.7±6.7 μM), M628L (23.1±0.07 μM), L795I (16.5±6.4 μM), and I796V (22.0±9.9 μM), suggesting that these three mutations do not directly contribute to changes in the recognition of the 4'ClMe moiety (Fig 4D). In contrast, the A789V mutation caused a moderate increase in the K_m_ of 4'ClMe-CTP (61.0±26.9 μM), although not as high as the K_m_ measured with the QUAD mutant (235±77 μM). The crystal structure of vesicular stomatitis virus (VSV) polymerase was used as a model to understand the molecular basis for the discrimination effect of the QUAD mutations in RSV polymerase [9]. A homology-based three dimensional structure of the RSV polymerase L protein was generated using RaptorX [10] (Fig 4E). The RSV polymerase model shows that out of the four mutated amino acids, only Ala789 (Asn692 in VSV, see sequence alignment in S1 Fig) is in close proximity to the nucleotide binding site as depicted by the GDNQ catalytic motif of the RdRp (Fig 4E).

### AZ-27 inhibits the RdRp function without dissociating the L-P subunits

**Fig 5:**
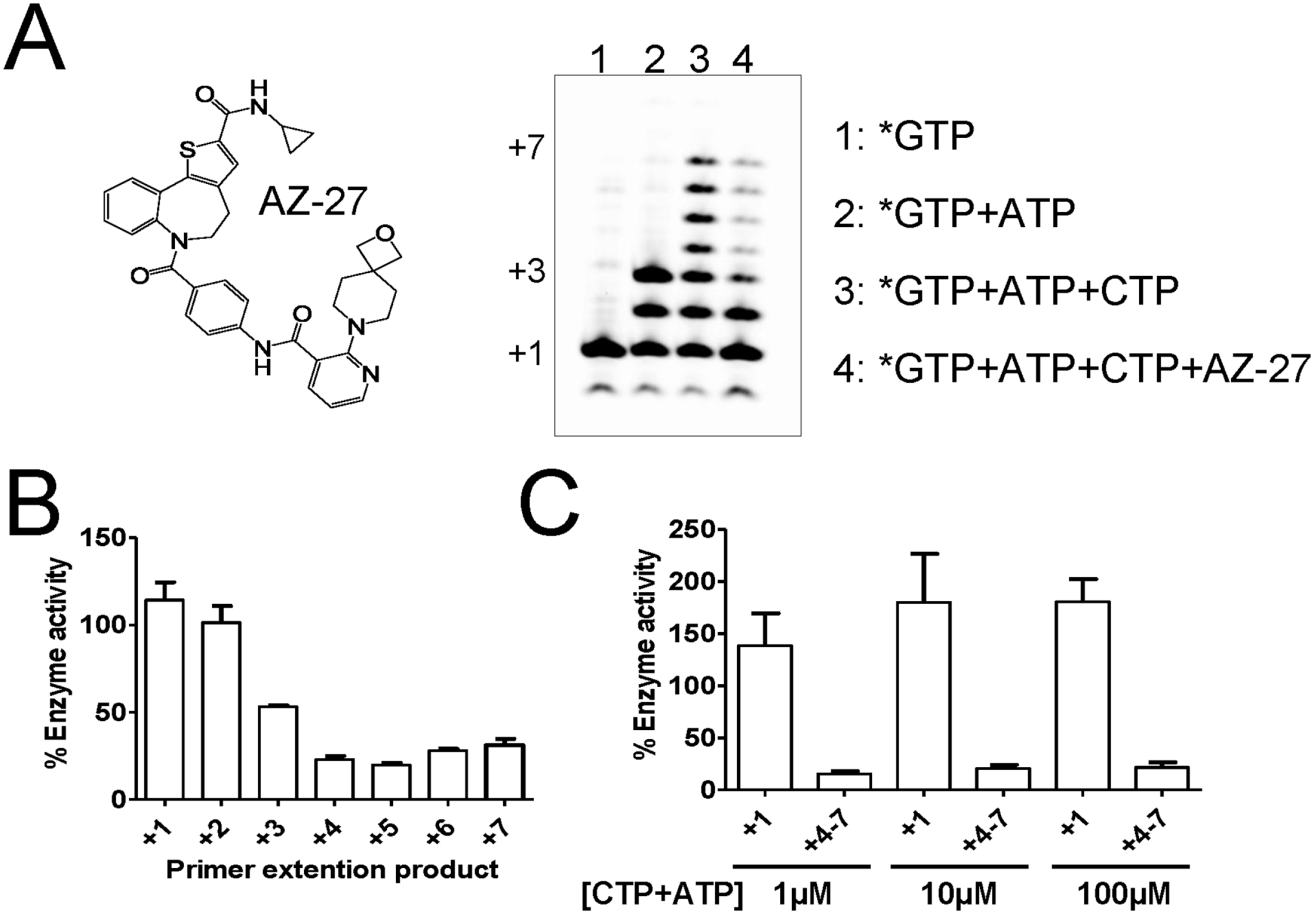
Inhibition of primer extension by AZ-27. **(A)** Standard WT RSV L-P primer extension assay with in Lane 1, *GTP; in Lane 2, *GTP+ATP; in Lane 3, *GTP+ATP+CTP; in Lane 4, *GTP+ATP+CTP+AZ-27. (**B**) Each individual primer extension RNA product (from +1 to +7) was quantified for Lane 4 (AZ-27), and compared with the corresponding amount of RNA in Lane 3 (no inhibitor). (**C**) The same experiment was repeated, using increasing concentrations of ATP+CTP from 1 to 100 μM (n = 2). The amount of +1 and +4–7 RNA products were measured in presence of AZ-27 at 10 μM, and compared with the corresponding amount of RNA without inhibitor (n = 2).

AZ-27 is a non-nucleoside RSV polymerase inhibitor believed to bind outside of the RdRp domain of the L protein (Fig 5A) [11,12]. The inhibition effect of AZ-27 on RSV polymerase was profiled in the standard primer-extension RdRp assay, as described in Fig 1. In the presence of *GTP and *GTP+ATP, RSV polymerase extended the 4-mer product specifically to the template sequence to reach a +1 and +3 product, respectively (Fig 5A, **lanes 1 and 2**). In the presence of *GTP+ATP+CTP, the 4-mer primer was further elongated to the +7 full-length extension product (Fig 5A, **lane 3**). Adding 10 μM of AZ-27 to the 30-minute enzymatic reaction resulted in only partial inhibition of primer extension. The amount of inhibition caused by AZ-27 changed for each RNA product size, varying from no inhibition at +1 and +2, to 75% to 85% inhibition at position +4 and beyond (see +4–7, Fig 5B). The same experiment was repeated using increasing concentrations of ATP+CTP from 1 to 100 μM (Fig 5C). The amount of +1 product from GTP incorporation remained high in the presence of 10 μM of AZ-27. As previously observed, the +4–7 RNA products were 75–85% inhibited irrespective of the concentration of ATP+CTP. This is indicative of non-competitive inhibition behavior.

**Fig 6:**
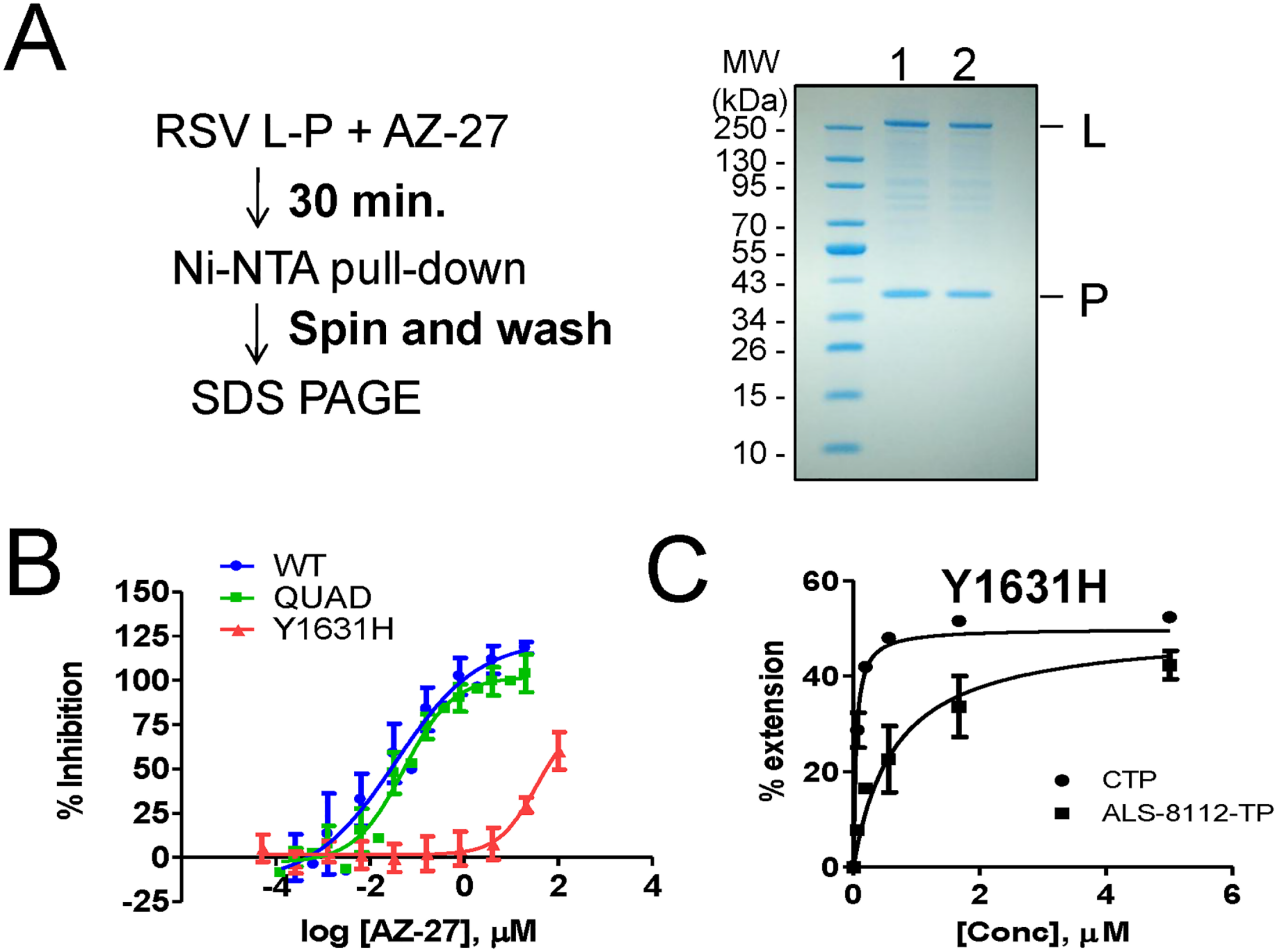
Loss of inhibition of AZ-27 with RSV polymerase Y1631H mutant. **(A)** RSV L-P pull-down assay. The enzyme-inhibitor complex (DMSO in Lane 1, and AZ-27 Lane 2) was pre-incubated for 30 minutes, after which nickel beads were used to pull down the histidine-tagged P subunit. The bound protein was washed on the beads and visualized by SDS PAGE. (**B**) Dose-response inhibition of RSV L-P RdRp in the biotin-primer extension assay. Reduction in the enzymatic activity of WT, QUAD, and Y1631H mutant was measured in the presence of increasing concentrations of AZ-27. (**C**) K_m_ determination for CTP and ALS-8112-TP by RSV polymerase containing the Y1631H mutation. K_m CTP_ = 0.042±0.009 μM (n = 4), and K_m ALS-8112-TP_ = 0.75±0.46 μM (n = 2).

A pull-down experiment was designed to understand if the inhibition effect of AZ-27 was caused by a dissociation of the RSV L-P dimer (Fig 6A). The enzyme-inhibitor complex was pre-incubated for 30 minutes, after which nickel beads were used to pull down the histidine-tagged P subunit. The bound protein was washed on the beads and visualized by SDS PAGE. When the experiment was performed in the presence of DMSO, the untagged L subunit remained noncovalently bound to P (Fig 6A, **lane 1**). Similarly, AZ-27 did not inhibit the ability of L to bind to P (Fig 6A, **lane 2**). We conclude that AZ-27 does not decrease the stability of the RSV L-P complex.

### Resistance of RSV polymerase mutants to AZ-27

The standard RSV polymerase primer-extension RdRp assay was slightly modified to specifically monitor +4 extension products by using a radiolabeled ^33^P-CTP tracer. A 4-mer primer containing a 5'-biotin label enabled high-affinity capture of all RNA products on streptavidin-coated 96-well plates, with unincorporated α^33^P-CTP removed by a simple wash step. In this assay format, the RdRp activity of WT RSV polymerase was inhibited with an IC_50_ value of 0.036 μM (Fig 6B). The QUAD RSV polymerase mutant had no significant effect on the inhibition potency of AZ-27, as judged by the unchanged IC_50_ value compared to the WT enzyme. In contrast, AZ-27 had an IC_50_ value of 34 μM against the RSV polymerase containing the Y1631H mutation in the L subunit, corresponding to a 940-fold resistance compared to the WT enzyme. We also measured the efficiency of nucleotide incorporation by Y1631H RSV polymerase. The K_m_ values for CTP and ALS-8112-TP were 0.042±0.009 and 0.75±0.46 μM, respectively, resulting in an 18-fold discrimination of the CTP analog (Fig 6C). Given that the discrimination of ALS-8112-TP by WT RSV polymerase is 13-fold [8], we conclude that the Y1631H mutation does not cause any significant decrease in the recognition of ALS-8112-TP. Taken together, these results indicate a lack of cross-resistance between nucleoside and non-nucleoside inhibitors associated with RSV polymerase mutations. The lack of cross-resistance effect between these two classes of inhibitors is explained by the fact that Y1631 is positioned in one of the capping domains, far away from the active site of the RdRp domain (S2 Fig).

### Antiviral effect of combining ALS-8112 with AZ-27

**Fig 7:**
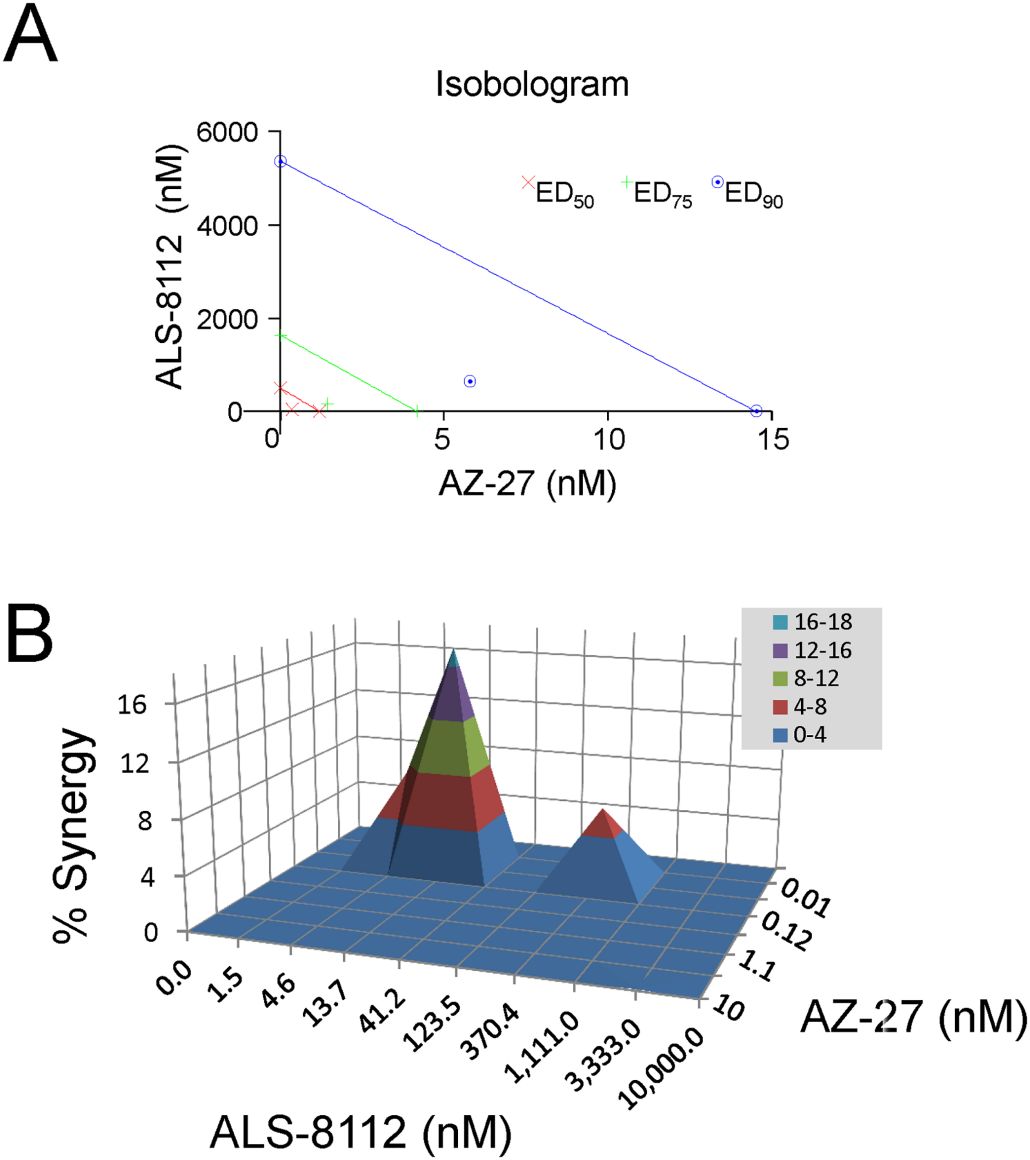
Synergistic antiviral effect of ALS-8112 and AZ-27 on RSV replication. (**A**) Isobologram analysis of ALS-8112 and AZ-27 interaction in HEp-2 cells against RSV replication. AZ-27 and ALS-8112 concentrations were on the x- and y- axis respectively. The three lines intersecting both axes represent additivity for EC_50_ (red), EC_75_ (green) and EC_90_ (blue). The calculated CI for EC_50_, EC_75_, and EC_90_ all fall below their respective additivity lines, indicating a synergy (0.3–0.7). (**B**) Prichard’s model for 3-D drug interaction dose-responses. The synergy volume was above the 0% plane and falls within 50–100 range, indicating a significant synergy (n = 5).

Antiviral combination studies were performed in vitro to measure the drug-drug interaction between ALS-8112 and AZ-27. HEp-2 cells were infected with recombinant RSV containing renilla luciferase as a reporter, in the presence of various concentrations of ALS-8112 and AZ-27 added individually or in combinations. Cell viability during the course of the experiment was >80% in all combinations of drug concentrations, indicating no increase in cytotoxicity when ALS-8112 and AZ-27 were combined together in HEp-2 cells (S3 Fig). The drug combination effect was first determined by the isobologram and combination index (CI) methods of Chou and Talalay [13,14], which provide quantitative estimation of the extent of synergy or antagonism. A CI of 1 indicates an additive effect between two compounds, whereas a CI< 1 or CI >1 indicates synergism or antagonism, respectively. With this analysis, the combination of ALS-8112 and AZ-27 resulted in significant synergy in antiviral effect, as determined by a CI = 0.38 for EC_50_, CI = 0.44 for EC_75_, and CI = 0.52 for EC_90_ (Fig 7A). Data were also analyzed using the Bliss-Independence model using Prichard’s MacSynergy II software [15]. This method uses a three-dimensional graphical layout representing the calculated additive surface predicting additive interaction as a horizontal plane at 0%. Peaks above this plane are indicative of synergy, and depressions below the horizontal plane indicative of antagonism. In the case of ALS-8112 combined with AZ-27, two peaks above the horizontal plane were observed with a total peak volume of 55.2 μM^2^% (Fig 7B). This volume is reflective of significant synergy of inhibition between the two drugs.

## Discussion

Currently there are no effective therapies or vaccines available for RSV infection (for recent review: [16]). The RNA polymerase of RSV is considered a major molecular target for therapeutic intervention because it plays a central role in the replication and transcription of the RSV genome. Like many other non-segmented negative sense (NNS) RNA viruses, the active form of the RSV polymerase is a hetero-dimer composed of the L and P proteins, L being the catalytic subunit responsible for the RdRp function [17]. In addition, it is believed that RSV polymerase is also responsible for mRNA cap addition and methylation, although this has only been experimentally demonstrated at the enzymatic level using the related VSV polymerase [18,19] [20].

One of our main goals was to understand, at the molecular level, the effect of inhibitor-induced resistance mutations on the function(s) of RSV polymerase. Two types of RSV polymerase inhibitors were evaluated: nucleoside and non-nucleoside analogs. The first part of the study was a follow-up to our initial work that elucidated the mechanism of RSV nucleotide resistance induced by the QUAD mutations [8]. Using recombinant RSV L-P protein, we previously showed that the QUAD mutations induced a 4.6-fold resistance to 2'F-4'ClCH_2_-CTP (ALS-8112-TP) in a single nucleotide incorporation assay. We now report a lack of cross-resistance when the 4'ClCH_2_ is changed to an azido (N_3_) group (Fig 1). This led us to evaluate a series of 2'F-4' substituted CTP analogs against QUAD L-P. Overall, the resistance phenotype was mainly associated with the presence of a 4'-halomethyl group, the highest level being observed with the bulky 4'BrCH_2_ group (Fig 2). Next, the standard primer extension assay was modified to accommodate single incorporation events of purine analogs. In these assays, both 2'F-4'ClCH_2_-GTP and 2'F-4'ClCH_2_-ATP acted as chain terminators (Fig 3). 2'F-4'ClCH_2_-GTP was strongly discriminated by the QUAD mutant with a 17-fold resistance level, whereas 2'F-4'ClCH_2_-ATP was incorporated with the same efficiency by WT and QUAD (Fig 3). This result reveals the importance of the nucleobase moiety for the discrimination of nucleotide analog by RSV polymerase. A similar phenomenon has been reported for hepatitis C virus (HCV) RNA polymerase, where the S282T mutation confers resistance against 2'F-2'CH_3_-UTP, but not against 2'F-2'CH_3_-GTP [21,22]. It would also be interesting to measure the potential excision of chain terminators and rescue of RNA synthesis by RSV polymerase since it has been reported previously for HIV and HCV polymerases [23,24]. Another aim of this study was to understand the individual contribution of the QUAD mutations to nucleotide resistance. For each of the three QUAD mutations present in motif B of the RdRp, individual L-P complexes containing a single amino acid change in the L protein were expressed and purified (Fig 4A). Because the level of resistance to 2'F-4'ClCH_2_-CTP is only 4.6-fold in the recombinant polymerase assay, K_m_ experiments were conducted instead with 4'ClCH_2_-CTP (Fig 4C, [8]). Among each of the four mutations, only A789V conferred some resistance, although not at the same level as with all four combined (Fig 4D). The contribution of A789V toward resistance to 4'ClCH_2_-substituted nucleotide analogs is supported by its predicted proximity to the modeled nucleotide binding site of the RdRp domain of RSV polymerase (Fig 4E). Our current hypothesis is that alanine 789 may be in close proximity of the ribose moiety of the incoming nucleotide during initial binding, catalysis, or both. In the absence of any high-resolution crystal structure of RSV L in complex with an NTP, the exact position and role of residue A789 remains unknown. More work will also be needed to fully understand the contribution of the three other QUAD mutations to nucleotide resistance. Also, a detailed description of the characterization of the ALS-8112 resistant virus and the role of each individual QUAD mutation on virus fitness will be the subject of a separate study. At the biochemical level, our data provide enough evidence to conclude that the QUAD mutations in RSV polymerase do not confer broad resistance to all nucleotide analogs, but instead show some level of specificity toward 4'-halomethyl cytidines and guanosines. This information could potentially be used to design novel nucleotide analogs that remain active against the QUAD RSV polymerase.

The second goal of the study was to understand the effect of Y1631H RSV L-P mutation on the interaction with RSV inhibitors. We used AZ-27 as a tool compound because it is so far the only known non-nucleoside inhibitor of recombinant RSV polymerase [12,25]. The Y1631H mutation has been associated with a strong resistance to AZ-27 and its parent molecule, YM-53403 [12,25]. Although the resistance phenotype is well characterized at the virus level, this effect has not been studied at the enzyme level. Because Tyr1631 is located outside the RdRp region of RSV polymerase (S3 Fig), there is a possibility that AZ-27 may interfere with one of the enzymatic steps required for mRNA capping. Even though the biochemical assays needed to address this hypothesis have been developed for VSV polymerase, the counterpart experiments conducted in our hands with recombinant RSV polymerase failed to detect any specific capping activity (data not shown). Instead, we present several lines of evidence to propose that AZ-27 is an allosteric inhibitor of the RdRp function of RSV polymerase acting by a non-competitive mechanism. First, we showed that AZ-27 inhibited primer extension in the gel-based RdRp assay (Fig 5A). The compound did not inhibit the first two nucleotide incorporation steps, with product inhibition only visible starting at position +3 on the elongated primer (Fig 5A and 5B). This effect was not base-specific, as demonstrated by the fact that the inhibition was non-competitive with respect to NTPs (Fig 5C). One alternative explanation could be that AZ-27 blocks a conformational change preventing the short primer from exiting the RdRp domain, although more work will be needed to further support this model. Allosteric inhibition of conformational change is a common mechanism of action for non-nucleoside inhibitors of viral polymerases, as exemplified by human immunodeficiency virus RT [26] and hepatitis C virus (HCV) NS5B [27–29]. In a recent study, it was found that allosteric inhibitors of HCV NS5B block a conformational change involved in the transition from initiation to elongation of primer synthesis [30], suggesting a common mode of action between these inhibitors and AZ-27. In the case of RSV polymerase, the protein must undergo multiple conformational changes to initiate primer synthesis, elongate, cap, and finally methylate the primer. Each transition from one step to the next represents a potential target for allosteric inhibition. Direct evidence of AZ-27 targeting the RdRp function of the L protein by allosteric mechanism was provided by the loss of inhibition potency in the presence of the Y1631H mutation (Fig 6B). However, the Y1631H mutation did not induce any change in the efficiency of incorporation of nucleotide analogs (Fig 6C). Conversely, the QUAD mutant containing amino acid changes near the active site of the RdRp domain remained fully susceptible to AZ-27 inhibition (Fig 6B).

Finally, the in vitro interaction between ALS-8112 and AZ-27 leading to potential synergy or antagonism was evaluated in drug combination antiviral assays. Two independent analyses were performed, using the isobologram and combination index determinations with the Chou-Talalay method [13,14], and the MacSynergy II software derived from the Bliss-Independence model [15]. In both cases, ALS-8112 and AZ-27 had significant synergistic effect on the inhibition of RSV replication (Fig 7). Importantly, there was no increase in cytotoxicity when ALS-8112 and AZ-27 were combined together in HEp-2 cells. This represents the first proof-of-concept study showing the positive impact of combining two different classes of RSV polymerase inhibitors. Further work will be needed to fully understand the extrapolation of these in vitro results to animal or human in vivo efficacy. As more RSV replication inhibitors progress toward clinical trial stage evaluation, synergistic effect between drugs and lack of broad cross-resistance induced by mutations in RSV polymerase will become strong arguments in favor of potential combination treatments.

## Materials and Methods

### Compounds

ALS-8112, ALS-8112-TP, their derivatives, and AZ-27 were all synthesized at Alios BioPharma (South San Francisco, CA, USA) according to described procedures [7,12]. Except for NTP analogs, all other compounds were stored at 4°C in dimethyl sulfoxide (DMSO). All the phosphorylated species were reconstituted in water, aliquoted, and stored at -80°C.

### Single nucleotide incorporation by recombinant RSV L-P dimer

The recombinant RSV polymerase complex was produced by co-expressing the L and P proteins of RSV in insect cells as previously described [31]. Unless otherwise specified, RNA polymerase reaction samples consisted of 0.2 μM of an oligonucleotide template sequence derived from the RSV leader promoter (5'-UUUGUUCGCGU-3') and 0.2 μM recombinant RSVL-P polymerase together with 200 μM 5'-pACGC primer, mixed in a buffer containing 20 mM Tris pH 7.5, 10 mM KCl, 2 mM dithiothreitol, 0.5% triton X-100, 10% DMSO, 0.2 U/μL RNasin (Ambion), and 6 mM MgCl_2_. Reactions were started by adding specific NTPs to a final volume of 10 μL and incubated at 30°C. The radioisotope tracer used for this assay was α^33^P-GTP. To measure the incorporation of GTP analogs, the oligonucleotide template sequence was modified to 5'-UUUCUUGGCGU-3'. Reactions were stopped after 30 minutes by adding an equal volume of gel loading buffer (Ambion). Samples were denatured at 95°C for 5 minutes, and run for 1.5 hours at 80 W in a 22.5% polyacrylamide urea sequencing gel. After the gel was dried, the product of migration was exposed to a phosphor-screen and scanned on a Typhoon phosphorimager (GE Healthcare) and quantified using ImageQuant (GE Healthcare). For each enzyme, the level of NTP analog discrimination was calculated as K_m NTP analog_ / K_m NTP_, and the corresponding resistance as Mutant_discrimination_ / WT_discrimination_.

### Ni-NTA pull-down assay

RSV L-P (8 μg) was incubated with DMSO or AZ-27 (10 μM) and 20 μL of pre-equilibrated Ni-NTA beads in buffer containing 20 mM Tris pH 7.5, 10 mM KCl, 6 mM MgCl_2_, 0.01% Triton X-100, 10% DMSO and 2 mM DTT in a total volume of 100 μL, for 30 minutes at 4°C with rocking. Beads were collected by centrifugation, and washed in 500 μL buffer containing 20mM Tris pH 7.5, 10 mM KCl, 6 mM MgCl_2_, 0.01% Triton X-100, and 2 mM DTT. Beads were resuspended in 20 μL loading buffer (4% SDS, 20% glycerol, 0.12M Tris pH 7, and 10% BME) and heated for 5 minutes at 95°C. Samples were run on a 4% to 12% SDS PAGE gel alongside PageRuler prestained marker (Invitrogen) and stained using SimplyBlue SafeStain (Thermo Fisher Scientific).

### Biotin-primer extension assay

Extension reactions contained 5 nM RSV polymerase L-P complex, incubated with 200 nM RNA (5'-UUUGUUCGCGU) and 4 μM 5' biotin labeled RNA primer (5'-biotin-ACGC), serial diluted inhibitor, mixed in a buffer with 20 mM Tris HCl pH 7.5, 10 mM KCl, 6 mM MgCl_2_, 0.01% Triton X-100, 10% DMSO, 2 mM DTT, 10 μM GTP, 10 μM ATP, and 25 nM α^33^P-CTP. 10 μL reactions were incubated at 30°C for 2 hours and stopped with the addition of EDTA. Stopped reactions were transferred to a Flashplate (Perkin Elmer), washed two times with 0.1% Tween-20 and read on a Microbeta Trilux.

### Cell line and recombinant RSV

HEp-2 cells were purchased from American Tissue Culture Collection and maintained in minimum essential medium (Corning) supplemented with 10% fetal bovine serum (Mediatech) and 1% penicillin/streptomycin (Mediatech). Cells were incubated at 37°C with 5% CO_2_.

Recombinant RSV containing a renilla luciferase reporter gene (A2-RL-line19F) was generated and previously described [32]. The virus contains a fusion protein of RSV line19 origin while all the other viral genes are in the A2 genetic background. In vitro replication of this recombinant virus is similar to A2 in HEp-2 cells [32].

### Drug combination study

HEp-2 cells were seeded into a 96-well plate at a 20,000 per well (100 μL) one day before compound dosing. ALS-8112 and AZ-27 were first serially diluted in DMSO to 200 folds of their respective final concentrations. Briefly, ALS-8112 was diluted to 9 concentrations horizontally and AZ-27 was diluted to 7 concentrations vertically, in separate 96-well plates. For each compound, a column or a row for single drug treatment was set up as the control. The first row in each 96-well plate was DMSO treatment control. 5 μL of each compound was then added into 90 μL MEM (cell plate) to achieve a combination matrix with 10-fold of final concentration. Finally, 10 μL was transferred from the cell plate to HEp-2 cells in 96-well plates for overnight incubation. The next day, A2-RL-line19F virus was added to the plate at a multiplicity of infection of 2 for two days before measuring the reporter activity using the Renilla Luciferase Assay System (Promega) on a Perkin Elmer multilabel counter Victor 3V. Five replicate experiments were performed and data analyzed.

### Cell viability assay

HEp-2 cells were seeded into a 96-well plate at 5,000 per well (100 μL). Compounds were added in the same format and concentrations as above except no virus was added afterwards. Cell viability was measured using an ATP-based firefly luciferase system, CellTiter-Glo^®^ (Promega). Luminescence was measured using a Perkin Elmer multilabel counter Victor3V. The relative percentage of firefly luciferase signal over the DMSO treatment control was plotted in 3-D contour. For each combination study, five experiment replicates were performed.

### Isobologram and MacSynergy analysis of drug interactions

The effects of combining two compounds were evaluated using the CalcuSyn (Biosoft, Ferguson, MO) based on the method of Chou and Talalay [13,14]. The software calculates a combination index (CI) at EC_50_, EC_75_ and EC_90_ using the Loewe additivity model. A CI of < 1, = 1, and > 1 indicates synergy, additivity, and antagonism, respectively. Under the synergy category, CI < 0.1 is considered very strong synergism; CI 0.1–0.3 strong synergism; CI 0.3–0.7 synergism, and CI 0.7–0.85 moderate synergism.

MacSynergy II software was used for three dimensional analysis of drug combination dose response surfaces using the Bliss-Independence model [15]. The model calculates a theoretical additive interaction from the dose response curves of each compound used individually. This calculated additive surface is then subtracted from the experimental dose-response surface to obtain the non-additive surface. The remaining surface would appear as a horizontal plane at 0%. Peaks above the 0% surface would indicate synergy whereas depressions in the plane indicated antagonism. The volume (μM^2^%) of the peak (or depression) was calculated at various significance levels and used to categorize the level of synergy (or antagonism). Volume of less than 25 indicates additivity, 25–50 minor synergy (or antagonism), 50–100 for significant synergy (or antagonism), >100 for strong synergy (or antagonism), and >1000 for probable errors.

### RSV minigenome assay

A modified RSV minigenome assay based on a previously described method [32,33] was used to assess the phenotypic shift in the quadruple mutation. In brief, HEp-2 cells were plated in 6-well plates at the density of 0.5 million cells/well. ALS-8112 was serially diluted and added to each of the wells and further incubated overnight. On the next day, modified vaccinia virus Ankara-T7 (MVA-T7) at the multiplicity of infection of 1 was added to provide T7 RNA polymerase [34]. After 2 hours of viral transduction, each well was transfected with Fugene 6 (Promega) with 1.25 μg mixture of 6 plasmids including minigenome (pGem.RSV.M5.Luc), plasmids encoding human codon bias-optimized N, P, M2-1, L genes as well as control plasmid encoding renilla luciferase pRL-SV40. After 48 hours of further incubation, the firefly luciferase as well as renilla luciferase signals from each well were measured with dual-luciferase reporter assay system (Promega). Sigmoidal dose-response curves used to generate 50% inhibitory or effective concentrations were analyzed by nonlinear regression using the four-parameter logistic equation (GraphPad Prism).

## Supporting Information

S1 Fig Amino acid sequence alignment between VSV and RSV L protein RdRp domains. The blue arrows represent the VSV amino acid equivalents of M628, A789, L795, and I796 on the RSV sequence.
(TIF)

S2 Fig Domain organization in RSV/VSV L protein. The structure of the L protein of VSV polymerase complex (PDB = 5a22, [9]) shows the RdRp domain in blue, and the domains involved in capping in green, yellow, red, and orange. The putative position in RSV of the Y1631H mutation within the capping region is circled. The QUAD mutations are located within the RdRp domain (first 900 amino acids), whereas the Y1631H mutation is positioned just upstream of the MTase domain.
(TIF)

S3 Fig Cell viability in drug interaction study. Cell viability as measured by quantifying ATP amount. Under all drug treatment concentrations, cell viability was at least 80% compared to DMSO treatment control cells. Data obtained from 5 experiment replicates.
(TIF)

## Abbreviations

CI: Combination index
EC_50_: Concentration resulting in 50% of the maximum effective response
IC_50_: Concentration resulting in 50% of the maximum inhibition
K_m_: Michaelis constant
NNS: Non-segmented negative sense
NTP: Nucleoside triphosphate
RdRp: RNA dependent RNA polymerase
RSV: Respiratory syncytial virus
RT: Reverse transcriptase
SD: Standard deviation
SEM: Standard error of the mean
TP: Triphosphate
VSV: Vesicular stomatitis virus
