# Supplementary figures and images for: Biochemical Effect of Resistance Mutations against Synergistic Inhibitors of RSV RNA Polymerase
Source: PLoS One. 2016 May 10;11(5):e0154097. doi: 10.1371/journal.pone.0154097 (PMC4862670; doi:10.1371/journal.pone.0154097)

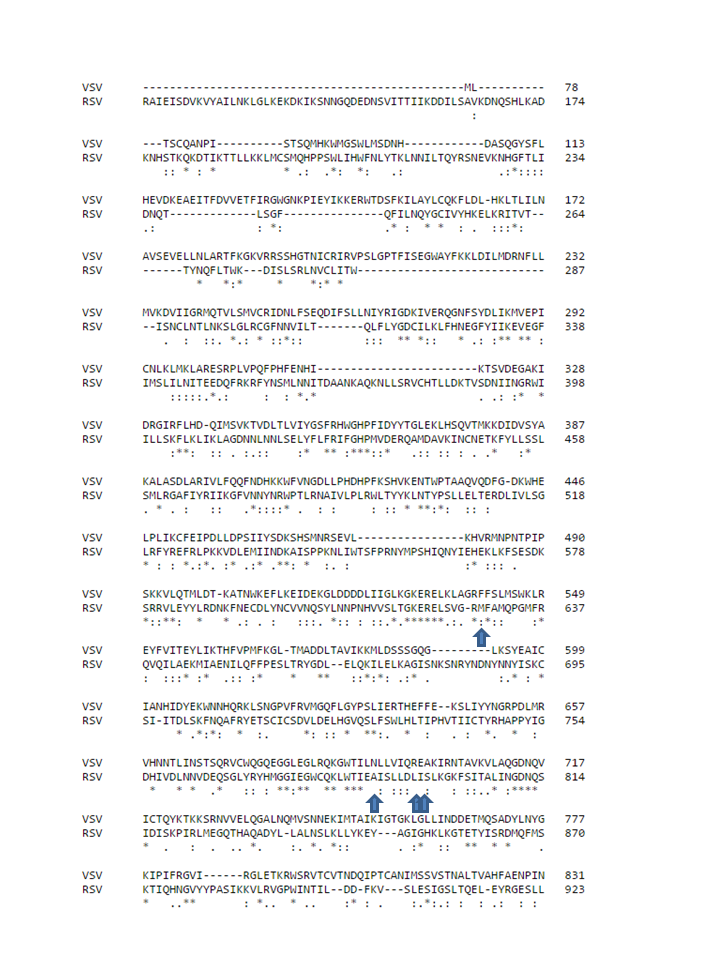

Supplement: S1 Fig — The blue arrows represent the VSV amino acid equivalents of M628, A789, L795, and I796 on the RSV sequence. (TIF) [file pone.0154097.s001.TIF]

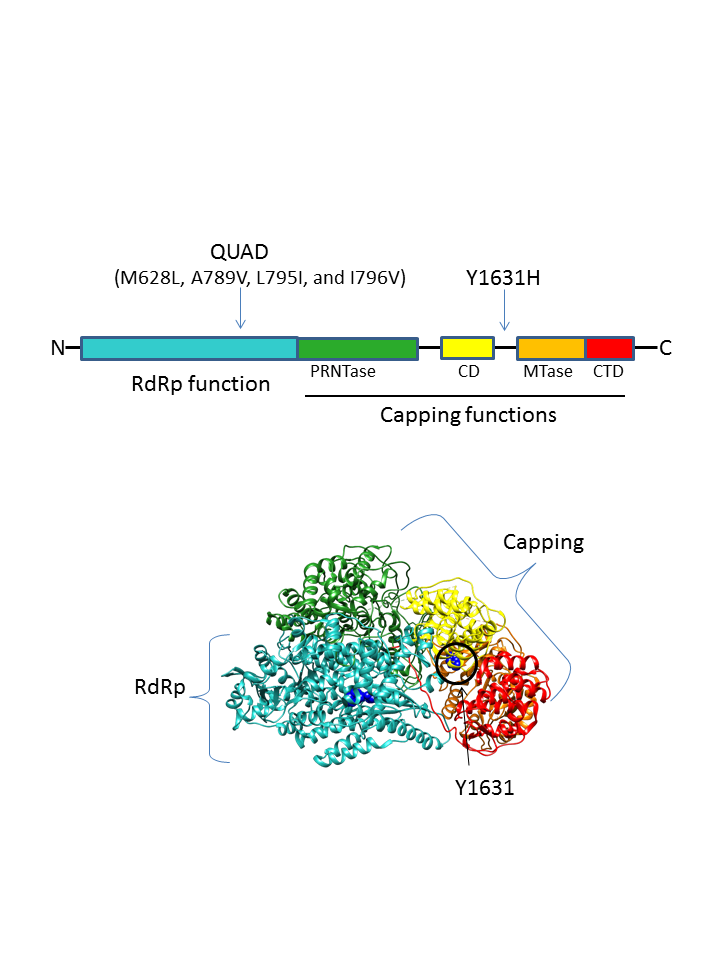

Supplement: S2 Fig — The structure of the L protein of VSV polymerase complex (PDB = 5a22, [9]) shows the RdRp domain in blue, and the domains involved in capping in green, yellow, red, and orange. The putative position in RSV of the Y1631H mutation within the capping region is circled. The QUAD mutations are located within the RdRp domain (first 900 amino acids), whereas the Y1631H mutation is positioned just upstream of the MTase domain. (TIF) [file pone.0154097.s002.TIF]

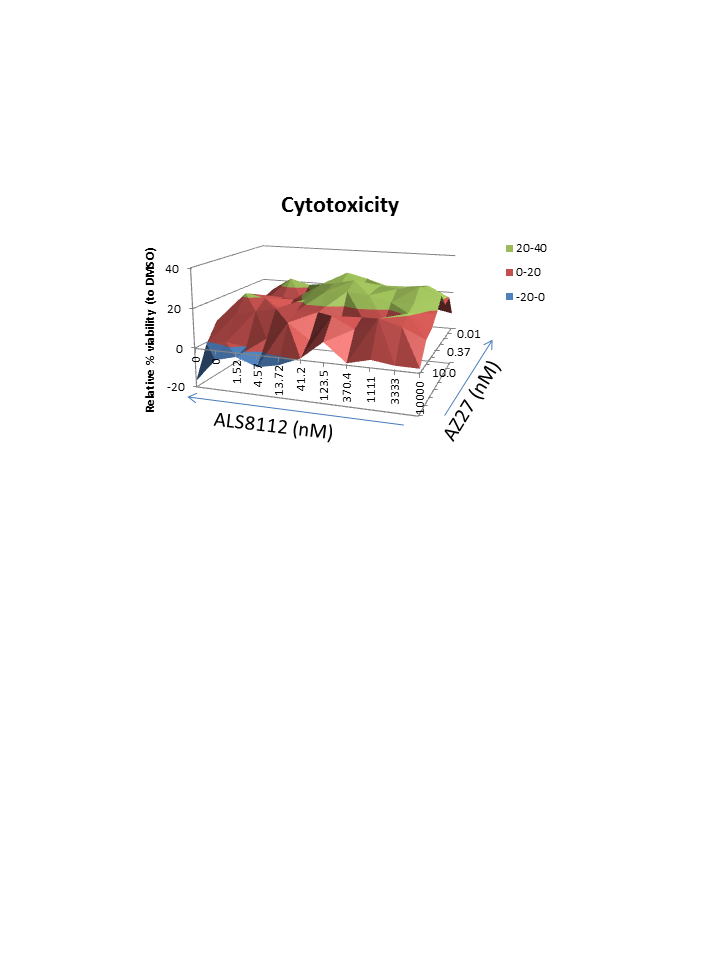

Supplement: S3 Fig — Cell viability as measured by quantifying ATP amount. Under all drug treatment concentrations, cell viability was at least 80% compared to DMSO treatment control cells. Data obtained from 5 experiment replicates. (TIF) [file pone.0154097.s003.TIF]
